# Genetically supported causality between gut microbiota, immune cells and morphine tolerance: a two-sample Mendelian randomization study

**DOI:** 10.3389/fmicb.2024.1343763

**Published:** 2024-02-08

**Authors:** Shuai Han, Jiapei Gao, Zi Wang, Yinggang Xiao, Yali Ge, Yongxin Liang, Ju Gao

**Affiliations:** ^1^Department of Anesthesiology, Northern Jiangsu People’s Hospital, Clinical Medical School, Yangzhou University, Yangzhou, China; ^2^Yangzhou University Medical College, Yangzhou, China; ^3^Department of Anesthesiology, Women’s and Children’s Hospital Affiliated to Qingdao University, Qingdao, China

**Keywords:** Mendelian, gut microbiota, immunity, morphine tolerance, causality

## Abstract

**Background:**

Previous researches have suggested a significant connection between the gut microbiota/immune cells and morphine tolerance (MT), but there is still uncertainty regarding their causal relationship. Hence, our objective is to inverstigate this causal association and reveal the impact of gut microbiota/immune cells on the risk of developing MT using a two-sample Mendelian randomization (MR) study.

**Methods:**

We conducted a comprehensive analysis using genome-wide association study (GWAS) summary statistics for gut microbiota, immune cells, and MT. The main approach employed was the inverse variance-weighted (IVW) method in MR. To assess horizontal pleiotropy and remove outlier single-nucleotide polymorphisms (SNPs), we utilized the Mendelian randomization pleiotropy residual sum and outlier (MR-PRESSO) technique as well as MR-Egger regression. Heterogeneity detection was performed using Cochran’s *Q*-test. Additionally, leave-one-out analysis was carried out to determine if any single SNP drove the causal association signals. Finally, we conducted a reverse MR to evaluate the potential of reverse causation.

**Results:**

We discovered that 6 gut microbial taxa and 16 immune cells were causally related to MT (*p* < 0.05). Among them, 2 bacterial features and 9 immunophenotypes retained a strong causal relationship with lower risk of MT: genus. *Lachnospiraceae NK4A136group* (OR: 0.962, 95% CI: 0.940–0.987, *p* = 0.030), genus. *RuminococcaceaeUCG011* (OR: 0.960, 95% CI: 0.946–0.976, *p* = 0.003), BAFF-R on B cell (OR: 0.972, 95% CI: 0.947–0.998, *p* = 0.013). Furthermore, 4 bacterial features and 7 immunophenotypes were identified to be significantly associated with MT risk: genus. *Flavonifractor* (OR: 1.044, 95% CI: 1.017–1.069, *p* = 0.029), genus. *Prevotella9* (OR: 1.054, 95% CI: 1.020–1.090, *p* = 0.037), B cell % CD3-lymphocyte (OR: 1.976, 95% CI: 1.027–1.129, *p* = 0.026). The Cochrane’s *Q* test revealed no heterogeneity (*p* > 0.05). Furthermore, the MR-Egger and MR-PRESSO analyses reveal no instances of horizontal pleiotropy (*p* > 0.05). Besides, leave-one-out analysis confirmed the robustness of MR results. After adding BMI to the multivariate MR analysis, the gut microbial taxa and immune cells exposure-outcome effect were attenuated.

**Conclusion:**

Our research confirm the potential link between gut microbiota and immune cells with MT, shedding light on the mechanism by which gut microbiota and immune cells may contribute to MT. These findings lay the groundwork for future investigations into targeted prevention strategies.

## 1 Introduction

Morphine exerts a potent analgesic effect primarily by interacting with mu opioid receptors (MOR). Its widespread utilization in pain management for conditions such as cancer is attributed to its rapid onset and prompt relief (Badshah et al., 2023). However, the chronic administration of morphine inevitably leads to various undesirable effects such as morphine tolerance (MT) and dependence, respiratory depression, nausea, vomiting, and sedation, which pose challenges to its clinical application (Ouyang et al., 2022). The development of morphine tolerance is a complex and multifaceted process, encompassing various factors such as the desensitization and internalization of opioid receptors (Narita et al., 2006), the formation of heterodimers between G protein-coupled receptors (Szentirmay et al., 2013), activation of glial cells, modifications in glutamate receptor function (Wang et al., 2021), stimulation of the cyclic adenosine monophosphate (cAMP) pathway (Coffey et al., 2022) and mitogen-activated protein kinase (MAPK) pathway (Fu and Zhang, 2023), among others. Considering the inadequate therapeutic outcomes and significant burden associated with morphine tolerance, it is imperative to identify potential underlying risk factors that contribute to its occurrence.

The human gut, which contains approximately 13 trillion bacterial cells, plays a crucial role in regulating various bodily functions such as vitamin production, colonization resistance, and immunological balance (Su et al., 2023). Recent research has suggested that the composition of gut microbiome may also be linked to conditions involving pain such as neuropathic pain, visceral pain and headaches (Minerbi and Shen, 2022). Studies have shown that morphine can disrupt the natural balance of bacteria in the gut leading to dysbiosis while also increasing intestinal permeability thereby facilitating bacterial translocation (Muchhala et al., 2023). The presence of microbial dysbiosis, specifically a reduction in *Bifidobacteria* and *Lactobacillaeae* populations, was observed in individuals undergoing MT. The administration of probiotics containing these bacterial communities demonstrated the ability to mitigate the development of analgesic tolerance in mice treated with morphine (Zhang et al., 2019). Additionally, recent studies have highlighted the significance of inflammatory factors released by immune cells in relation to MT (Eidson and Murphy, 2019). There is a growing body of research indicating that morphine has the potential to induce neuroinflammation (Wan et al., 2022). This process involves the activation of microglia (Mélik Parsadaniantz et al., 2015) and an increase in the production of proinflammatory cytokines, including IL-1β, TNF-α, CXCL12, IL-33, and IL-6 (Eidson et al., 2017; Bai et al., 2022), leading to heightened synaptic transmission and central sensitization (He et al., 2019). However, further investigation is needed to establish a definitive link between gut microbiota, immune cells, and the risk of MT due to possible biases.

Mendelian randomization (MR) is a highly effective approach to infer causality, employing genetic variants as instrumental variables (IVs) to investigate the causal impact of exposure on outcome (Wang et al., 2023). In this study, gut microbiota and immune cells were chosen as the exposure variables, while MT was considered as the outcome variable for MR analysis. The aim was to explore potential causal relationships and establish a theoretical foundation for further investigations into the intricate mechanisms and risk factors associated with MT.

## 2 Materials and methods

### 2.1 Ethics approval statement

The data at a summary level utilized in this research can be downloaded. The genome-wide association studies (GWAS) conducted for this study were approved by the appropriate institutions following ethical guidelines.

### 2.2 Study design

**Figure 1:**
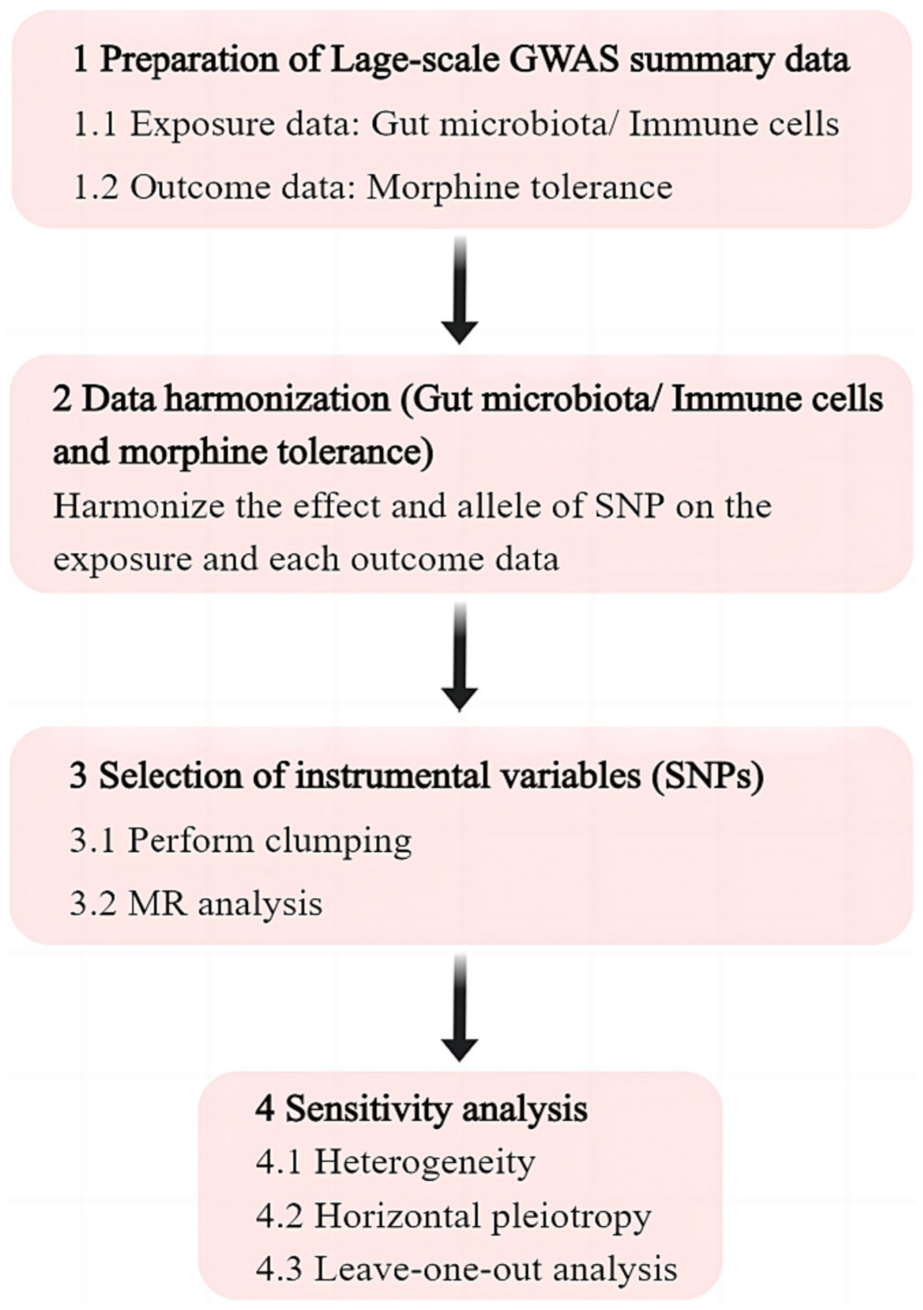
Flowchart of the study. GWAS, genome wide association study; SNP, single nucleotide polymorphism; MR, Mendelian randomization.

The exposure for investigation were gut microbiota and immune cells, whereas the outcome was MT. All data used in the analysis were obtained from publicly accessible GWAS. We extracted single nucleotide polymorphisms (SNPs) that are linked to gut microbial taxa and immune cells, which were then utilized as IVs. We performed a two-sample M analysis using summary level data from GWAS of gut microbiota, immune cells, and MT. The flow chart of this study is shown in Figure 1.

### 2.3 Exposure data of gut microbiota and immune cells

Relevant data on gut microbiota were extracted from a comprehensive GWAS study conducted by the MiBioGen consortium. This study involved 18,340 individuals (24 cohorts) and utilized 16S rRNA gene sequencing profiles (Kurilshikov et al., 2021). The analysis focused on 211 taxa, including 131 genera, 35 families, 20 orders, 16 classes, and 9 phyla for mapping microbiome quantitative trait loci. Information regarding each immune trait’s summary statistics was publicly accessible through the GWAS Catalog with accession numbers ranging from GCST0001391 to GCST0002121 (Orrù et al., 2020). A total of 731 immunophenotypes were analyzed, encompassing various parameters such as absolute cell (AC) counts (*n* = 118), median fluorescence intensities (MFI) indicating surface antigen levels (n = 389), morphological parameters (MP) (*n* = 32), and relative cell counts (RC) (*n* = 192). The MFI, AC, and RC features comprise B cells, CDCs, mature T cell stages, monocytes, myeloid cells, TBNK panels (T cells, B cells, natural killer cells), and Treg panels. Additionally, the MP feature includes CDC and TBNK panels. The initial GWAS analysis focused on immune traits and involved a sample of 3,757 individuals of European descent. The cohorts used in the study did not overlap. To enhance the genotyping data, around 22 million SNPs were imputed using a Sardinian sequence-based reference panel (Sidore et al., 2015). Additionally, associations were examined while accounting for potential confounding factors such as sex, age, and age squared.

### 2.4 Outcome data of morphine tolerance

The GWAS summary statistics for genetic associations related to MT were obtained from the largest GWAS meta-analysis conducted by the Neale Lab (Neale, 2016). This study involved 337,159 individuals of European descent (194 cases and 336,965 controls), with analysis performed on approximately 10.8 million variants following quality control measures and imputation.

### 2.5 Genetic instruments selection and harmonization

To ensure the reliability and precision of outcomes, we conducted a quality check on the SNPs to obtain compliant IVs. The selection criteria for SNPs were as follows: (A) strong association with exposures; (B) no correlation with confounding factors; (C) association with outcomes influenced by the exposures (Burgess et al., 2019). As the number of eligible IVs (genome-wide statistical significance threshold, *p* < 5 × 10^−8^) was extremely limited, we opted for a locus-wide significance threshold (*p* < 1 × 10^−5^) to ensure a more comprehensive outcome (Lv et al., 2021). Subsequently, in order to address linkage disequilibrium (LD), we employed a clumping method with *r*^2^ = 0.001 and kb = 10,000. Finally, we computed the F statistics to evaluate the robustness of the chosen SNPs using the following equation: *F* = *R*^2^(*N* − *k* − 1)/[(1 − *R*^2^)*k*]. In this equation, *R*^2^ represents the proportion of variability explained by each SNP, *N* denotes the size of our GWAS sample, and *k* indicates the number of SNPs. A value of 10 for the *F* statistic suggests a lack of substantial evidence for instrument bias (Yengo et al., 2018).

### 2.6 Multivariate MR analysis

Obesity has recently emerged as a major confounding factor in the association of intestinal diseases, as it is intricately linked to the health outcome under investigation while potentially influencing the composition of the microbiome (Vujkovic-Cvijin et al., 2020). To address this concern and mitigate potential biases arising from sample overlap, we conducted multivariate Mendelian randomization (MVMR) analysis as a sensitivity analysis to account for measured confounders, with body mass index [BMI, (SD, ~4.8 kg/m^2^)] being considered as a potential confounder. We utilized publicly available GWAS meta-analyses for BMI with a substantial sample size (Locke et al., 2015). The inverse-variance weighted method was employed for MVMR analysis.

### 2.7 Statistical analysis

The primary analysis for MR employed the inverse variance weighted (IVW) method. To evaluate the reliability of significant findings, sensitivity analyses were conducted using MR-Egger, weighted median, weighted mode, and simple mode (Hartwig et al., 2017). Heterogeneity among selected IVs was assessed by Cochran’s *Q* statistic and corresponding *p*-values. If the null hypothesis is not supported, random effects IVW was employed instead of fixed-effects IVW. To account for potential horizontal pleiotropy, a commonly used approach (MR-Egger) was applied, which suggests the presence of horizontal multiplicity if its intercept term is statistically significant. Additionally, we utilized the MR-PRESSO method from the MR-PRESSO package (Verbanck et al., 2018), a robust technique that helps identify and exclude any potential outliers with horizontal pleiotropic effects that could substantially influence our estimation results. Furthermore, scatter plots and funnel plots were utilized to assess the data. The scatter plots indicated that outliers did not have a significant impact on the results. Meanwhile, the funnel plots demonstrated that there was no heterogeneity and confirmed the robustness of the correlation. Additionally, a reverse causality analysis was conducted to evaluate any potential reverse causal relationships. All analyses were performed using R version 4.3.1 with packages such as “TwoSampleMR,” “MRPRESSO,” and “MendelianRandomization.” The codes used for these analyses can be found in Supplementary Table S1.

## 3 Results

### 3.1 Selection of instrumental variables

Initially, a total of 13,749 SNPs (gut microbiota; locus-wide significance level, *p* < 1 × 10^−5^) and 18,620 SNPs (immune cells; locus-wide significance level, *p* < 1 × 10^−5^) were identified as potential instrumental variables (IVs) from large-scale GWAS. These SNPs were selected after excluding palindromic variants (Supplementary Tables S2, S3). After clumping and harmonization, a total of 1,515 SNPs (*p* < 1 × 10^−5^) and 18,620 SNPs (*p* < 1 × 10^−5^) were identified as instrumental variables. The *F*-statistics for these IVs consistently exceeded the threshold of 10, indicating no indications of weak instrument bias. Detailed information on the key characteristics of the SNPs such as effect allele, alternate allele, beta value, standard error, and *p*-value was systematically collected for further analysis (Supplementary Tables S4, S5).

### 3.2 Causal effects of gut microbiota on morphine tolerance

**Figure 2:**
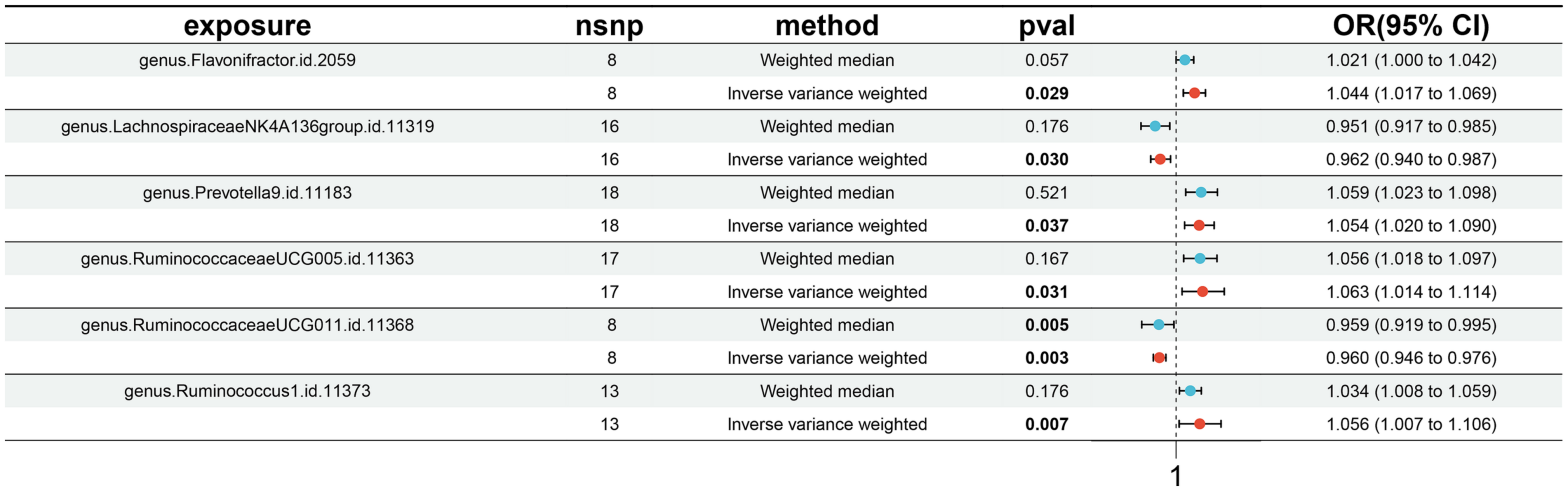
Forest plots presenting the Mendelian randomization findings of gut microbiota taxa associated with morphine tolerance causation. OR, odds ratio; CI, confidence interval; IVW, inverse variance weighted.

A total of six causal associations between gut microbiota features and MT traits were detected using the IVW method (Supplementary Table S6). The results from IVW analysis suggested that an increased genetic predisposition to the certain genus. *Flavonifractor* (OR: 1.044, 95% CI: 1.017–1.069, *p* = 0.029), genus. *Prevotella9* (OR: 1.054, 95% CI: 1.020–1.090, *p* = 0.037), genus. *RuminococcaceaeUCG005* (OR: 1.063, 95% CI: 1.014–1.114, *p* = 0.031), genus. *Ruminococcus1* (OR: 1.056, 95% CI: 1.007–1.106, *p* = 0.007) were linked to an elevated risk of MT. In addition, a lower risk of MT was observed in relation to the genetically predicted abundance of genus. *LachnospiraceaeNK4A136group* (OR: 0.962, 95% CI: 0.940–0.987, *p* = 0.030), genus. *RuminococcaceaeUCG011* (OR: 0.960, 95% CI: 0.946–0.976, *p* = 0.003) (Figure 2).

### 3.3 Causal effects of immune cells on morphine tolerance

**Figure 3:**
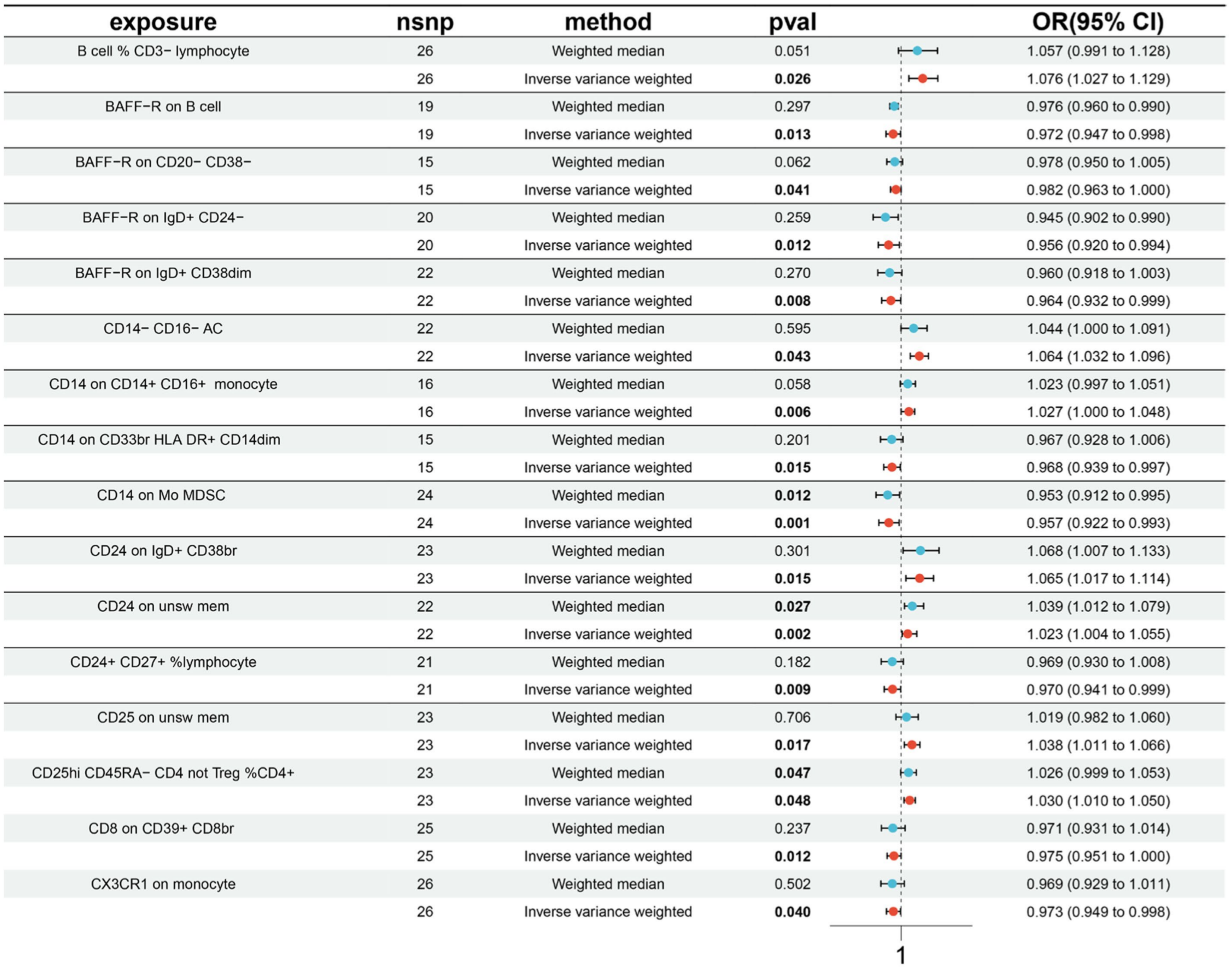
Forest plots presenting the Mendelian randomization findings on immune cells associated with morphine tolerance causality. OR, odds ratio; CI, confidence interval; IVW, inverse variance weighted.

A total of 16 causal associations between immune cell features and MT traits were discovered using the IVW method, with 8 in the B cell panel, 1 in the TBNK panel, 2 in the Treg panel, 3 in the monocyte panel, and 2 in the myeloid cell panel (Supplementary Table S7). The findings from the IVW analyses indicated that an increased genetic abundance of B cell % CD3− lymphocyte (OR: 1.976, 95% CI: 1.027–1.129, *p* = 0.026), CD14^−^ CD16^−^ AC (OR: 1.064, 95% CI: 1.032–1.096, *p* = 0.043), CD14 on CD14^+^ CD16^+^ monocyte (OR: 1.027, 95% CI: 1.000–1.048, *p* = 0.006), CD24 on IgD^+^ CD38br (OR: 1.065, 95% CI: 1.017–1.114, *p* = 0.015), CD24 on unsw mem (OR: 1.023, 95% CI: 1.004–1.055, *p* = 0.002), CD25 on unsw mem (OR: 1.038, 95% CI: 1.011–1.066, *p* = 0.017), CD25hi CD45RA-CD4 not Treg %CD4^+^ (OR: 1.030, 95% CI: 1.010–1.050, *p* = 0.048) were associated with a higher risk of MT. In addition, the genetically predicted abundance of BAFF-R on B cell (OR: 0.972, 95% CI: 0.947–0.998, *p* = 0.013), BAFF-R on CD20^−^ CD38^−^ (OR: 0.982, 95% CI: 0.963–1.000, *p* = 0.041), BAFF-R on IgD^+^ CD24− (OR: 0.956, 95% CI: 0.920–0.994, *p* = 0.012), BAFF-R on IgD^+^ CD38dim (OR: 0.964, 95% CI: 0.932–0.999, *p* = 0.008), CD8 on CD39^+^ CD8br (OR: 0.975, 95% CI: 0.951–1.000, *p* = 0.012), CD14 on CD33br HLA DR^+^ CD14dim (OR: 0.968, 95% CI: 0.939–0.997, *p* = 0.015), CD14 on Mo MDSC (OR: 0.957, 95% CI: 0.922–0.993, *p* = 0.001), CD24^+^ CD27^+^ %lymphocyte (OR: 0.970, 95% CI: 0.941–0.999, *p* = 0.009), CX3CR1 on monocyte (OR: 0.973, 95% CI: 0.949–0.998, *p* = 0.040) were correlated with a reduced risk of MT (Figure 3).

### 3.4 Pleiotropy, heterogeneity, sensitivity and reverse analysis

The results of the IVW test and MR-Egger regression indicated no heterogeneity in the majority of causal relationships, as suggested by *Q* statistics (*p* > 0.05) (Supplementary Tables S8, S9). Moreover, none of the intercepts derived from the MR-Egger regression analysis significantly differed from zero, suggesting no indication of horizontal pleiotropy (all intercept *p* > 0.05) (Supplementary Tables S10, S11). The MR-PRESSO test did not reveal any indications of horizontal pleiotropy in the examined causal relationships (*p* > 0.05) (Supplementary Table S12). Moreover, the Leave-one-out analysis demonstrated that individual SNPs did not significantly influence the signals associated with causality (Supplementary Tables S13, S14). Furthermore, during reverse MR analysis, no supportive evidence was found for a causal impact of MT on gut microbiota/immune cells (Supplementary Tables S15, S16).

### 3.5 Exploration of BMI as potential confounding factor

Obesity has recently been identified as a major confounding factor in the associations between microbiome and diseases. We conduct a multivariable MR analysis to assess the robustness of causal effects when incorporating obesity. After adjusted for BMI, the IVW results of MVMR analyses demonstrated that genus. *Flavonifractor* (OR: 1.051, 95% CI: 1.010–1.094, *p* = 0.013), genus. *RuminococcaceaeUCG005* (OR: 1.067, 95% CI: 1.020–1.124, *p* = 0.018), BAFF-R on B cell (OR: 0.932, 95% CI: 0.826–0.987, *p* = 0.021), BAFF-R on CD20^−^ CD38^−^ (OR: 0.901, 95% CI: 0.816–0.994, *p* = 0.038), CD14^−^ CD16^−^ AC (OR: 1.073, 95% CI: 1.022–1.115, *p* = 0.017), CD24 on IgD^+^ CD38br (OR: 1.127, 95% CI: 1.051–1.209, *p* = 0.015), CD8 on CD39^+^ CD8br (OR: 0.900, 95% CI: 0.815–0.993, *p* = 0.045), CD14 on CD14^+^ CD16^+^ monocyte (OR: 1.064, 95% CI: 1.0023–1.117, *p* = 0.014), and CX3CR1 on monocyte (OR: 0.954, 95% CI: 0.848–0.999, *p* = 0.019) were significantly correlated with the risk of MT. However, the remaining associations found may be confounded to some extent by BMI (Supplementary Table S17).

## 4 Discussion

**Figure 4:**
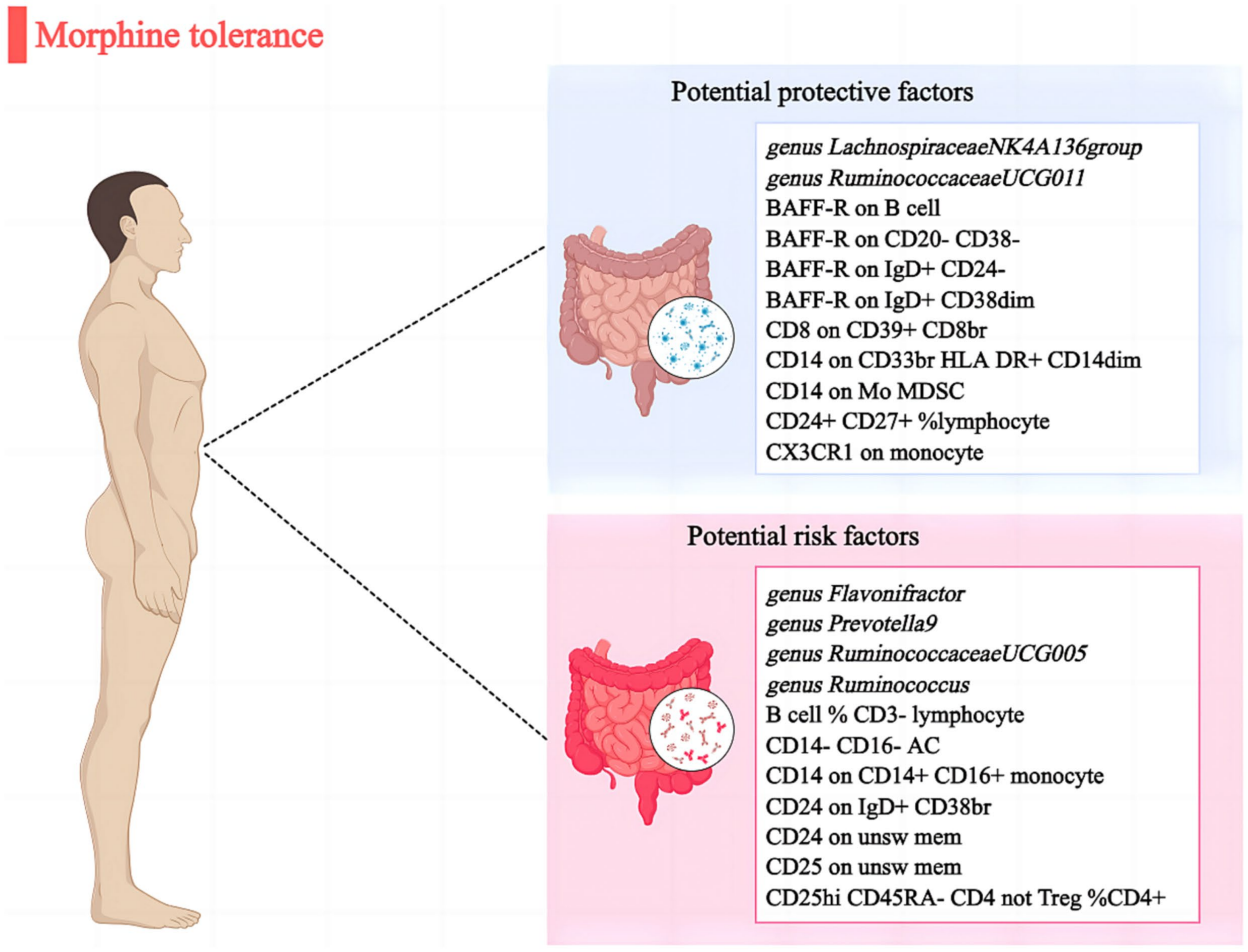
Causal links between gut microbiota, immune cells and morphine tolerance.

To our understanding, this MR analysis report appears to be the initial study establishing a potential causal link between gut microbiota/immune cells and MT. Through a two-sample MR investigation, we have discovered that there is evidence of causal correlation between 6 specific gut microbial taxa and 16 immune cells with regards to MT (Figure 4). Nevertheless, when BMI was included in the MVMR analyses, the impact of these factors was diminished.

Previous studies have extensively discussed the potential mechanisms by which the gut microbiota contributes to the development of MT. Disruption of the gut barrier and translocation of bacteria are observed as a result of morphine-induced dysbiosis, leading to local inflammation in the gut and subsequent activation of proinflammatory cytokines, ultimately driving MT. Both animal experiments and human studies have provided evidence for morphine’s detrimental effects on intestinal microbial composition, which further contribute to gastrointestinal disorders experienced by patients (Wang et al., 2018). The introduction of subcutaneous morphine granules or intraperitoneal morphine injections in mice resulted in an increase harmful bacteria, such as *Flavobacterium, Enterococcus*, *Fusobacterium*, *Sutterella, Clostridium*, *Rikenellaceae* and *Ruminococcus*, observed in fecal samples (Lee et al., 2018). Similarly, prolonged administration of morphine to mice led to alterations in the bacterial composition found in fecal samples. This included a decrease in *Bacteroidetes* and *Firmicutes* populations alongside an increase in *Proteobacteria* (Kang et al., 2017). Furthermore, there were reported elevations specifically within the phylum *Firmicutes* (Banerjee et al., 2016). Moreover, mice that developed tolerance to morphine exhibited a significant reduction in beneficial bacteria from the *Lactobacillus* and *Bifidobacterium*. However, this tolerance was mitigated following treatment with probiotics (Zhang et al., 2019). These studies indicate that chronic administration of morphine can lead to imbalances in the composition of gut bacteria. The close association between reductions in bacteria such as *Lactobacillus* or *Bifidobacterium* at the intestinal level and the development of dysbiosis emphasizes their crucial role in maintaining the integrity of the intestinal epithelium (Guo et al., 2017). Limited clinical research has been conducted to explore the connection between dysbiosis and the opioid system. A study involving cirrhotic patients who received long-term opioid treatment revealed a reduction in *Bacteroidaceae* relative abundance among those diagnosed with hepatic encephalopathy (HE). This decrease coincided with lower levels of aromatic amino acids and metabolites from specific bacterial strains while also showing elevated endotoxin and IL-6 levels (Acharya et al., 2017). Our findings align with prior research on the beneficial effects exerted by *Bacteroidaceae*. Furthermore, our investigation suggests that an increased presence of *Flavonifractor, Prevotella9, RuminococcaceaeUCG005*, *Ruminococcus1* is linked to an augmented risk for MT.

The expression levels of opioid receptors have been found to be associated with the composition of the gut microbiota. For instance, there is a positive correlation between the abundance of *Bacteroides* spp. in fecal matter and MOR mRNA in colonic tissue among rats (Aguilera et al., 2013). The chronic administration of morphine induces changes in intestinal epithelial function, resulting in increased permeability and bacterial translocation mediated by Toll-like receptor (TLR) (Meng et al., 2013). However, these effects were mitigated when mice were treated with a combination of antibiotics. In addition, the impact of opioids on the intestinal microbiota could worsen the effects of opioids themselves by causing inflammation. In a mouse model of sepsis, it was observed that blocking IL-17A after morphine treatment, which increases levels of Gram-positive bacteria, led to improved inflammation and function of the intestinal barrier (Meng et al., 2015). Administering a probiotic like *Lactobacillus acidophilus* in rats with irritable bowel syndrome (IBS) increased their pain threshold and alleviated hypersensitivity. This was achieved by activating the inflammatory NF-кB pathway to enhance MOR expression (Rousseaux et al., 2007).

Fecal microbiota transplantation (FMT) is currently being investigated as a potential approach to alleviate morphine tolerance in rodents with opioid-induced dysbiosis (Muchhala et al., 2023). Prolonged exposure to morphine reduces the presence of Regenerating islet-derived 3 gamma (Reg3γ), an antimicrobial peptide, in the ileum. This leads to decreased intestinal antimicrobial activity against Gram-positive bacteria like *L. reuteri*. However, when FMT was performed using fecal samples from mice not exposed to morphine, it restored the antimicrobial activity and expression of Reg3γ in the intestine. Consequently, this prevented an increase in intestinal permeability and the development of antinociceptive tolerance in mice dependent on morphine. *In vitro* strains, such as *Staphyloccocus aureus*, *S. epidermidis*, *Streptoccocus pneumoniae*, *S. pyogenes*, *S. faecalis*, *Bacillus cereus*, *Escherichia coli*, and *Pseudomonas aeruginosa* showed no growth inhibition upon exposure to a 2 mg/mL concentration of morphine (Rosenberg and Renkonen, 1985). In contrast, the growth of pathogenic bacteria in culture, such as *E. coli* and *S. epidermidis*, could be completely inhibited by tramadol at a concentration of 25 mg/mL (Farzam et al., 2018). In accordance with the aforementioned research, our study indicated a causal link between a decreased risk of MT and the heightened prevalence of *Lachnospiraceae* and *RuminococcaceaeUCG011*.

On the contrary, MT is also influenced by immune cells in its occurrence and development (Zhang et al., 2022). Extensive research has been dedicated to understanding the molecular mechanism behind morphine tolerance in recent years. A growing body of evidence supports the notion that morphine can initiate neuroinflammation, characterized by microglia activation and increased production of pro-inflammatory cytokines like tumor necrosis factor-α (TNF-α), Interleukin (IL)-6, and IL-1β. This subsequently exacerbates neuroinflammation, ultimately leading to MT (Guan et al., 2023).

Our research discovered the risk of morphine tolerance decreased with an increase in the proportion of BAFF-R on B cell, BAFF-R on CD20^−^ CD38^−^, BAFF-R on IgD^+^ CD24^−^, BAFF-R on IgD^+^ CD38dim, CD8 on CD39^+^ CD8br, CD14 on CD33br HLA DR^+^ CD14dim, CD14 on Mo MDSC, CD24^+^ CD27^+^ %lymphocyte, CX3CR1 on monocyte. B lymphocyte stimulator (BLyS) is a member of the tumor necrosis factor superfamily and plays a critical role in the proliferation, maturation, and differentiation of B lymphocytes. BAFF-R has the ability to impede the interaction between soluble BLyS and receptors on cell membranes, thereby inhibiting NF-κB activation and suppressing immunoglobulin production by B cells in peripheral blood (Xu and Chen, 2017). The increased levels of B-cell activating factor (BAFF) are a consequence of both acute and chronic inflammation (Matharu et al., 2013). Research findings indicate that the inhibition of the TLR4/NF-κB signaling pathway can effectively suppress microglia activation and neuroinflammatory responses, leading to an improved analgesic effect of morphine (Yang et al., 2023). HLA-DR, a cell surface receptor belonging to the MHC class II family, is encoded by the human leukocyte antigen complex located in chromosome 6 region 6P21. The reduced expression of HLA-DR in monocytes during chronic inflammation highlights its anti-inflammatory properties (Söderlund et al., 2009).

Furthermore, it is worth mentioning that the occurrence of MT was observed to be linked with a rise in the percentage of B cell % CD3^−^ lymphocyte, CD14^−^ CD16^−^ AC, CD14 on CD14^+^ CD16^+^ monocyte, CD24 on IgD^+^ CD38br, CD24 on unsw mem, CD25 on unsw mem, CD25hi CD45RA-CD4 not Treg %CD4^+^. Based on the differential expression of surface markers CD14 and CD16, monocytes can be categorized into two subgroups: CD14^+^ CD16^−^ and CD14^+^ CD16^+^. Notably, elevated levels of CD14^+^ CD16^+^ have been observed in conditions such as inflammatory pain and ANCA-associated vasculitis, leading to their identification as inflammatory monocytes. Research suggests that this subgroup’s mechanism of action may involve increased expression of relevant inflammatory receptors along with pro-inflammatory microRNAs (Xia et al., 2021). Based on the differential expression of CD25 on CD4^+^ T cells, they can be categorized into two groups: effector T cells (Teff) with low CD25 expression and regulatory T cells (Treg) with high CD25 expression. Both types can be activated through antigen stimulation via the signaling pathway of the T cell receptor (TCR). However, while Teff promotes immune inflammatory reactions, Treg maintains tolerance in the body, exhibiting distinct functions and roles. Morphine has been found to enhance the expression of several crucial pro-inflammatory chemokines as well as C-C Motif Chemokine Receptor 5 (CCR5) on T cells, monocytes, and macrophages. Moreover, morphine has the ability to elevate IL-10 levels while simultaneously reducing IL-17 secretion from cultured CD4^+^ T cells (Shao et al., 2022). Previous studies have reported a significant increase in the numbers of circulating Treg cells and the functional activity of Th17 cells following chronic exposure to morphine. Additionally, there is an observed increase in T cell populations expressing surface markers associated with gut-homing (CD161 and CCR6) as well as susceptibility to HIV-1 infection (CCR5 and β7 integrin) (Cornwell et al., 2013). However, limited research has been conducted on the role of monocytes and T cells in MT.

The advantages of this study are as follows: MR utilizes genetic variants as proxies for environmental exposure in order to establish a causal association between an exposure and the occurrence of a disease. Given that genetic variations are presumed to be randomly determined prior to birth, they exhibit a strong independence from environmental factors and become firmly established long before the onset of illness. This characteristic helps circumvent issues related to residual confounding and reverse causation commonly encountered in conventional observational studies. This research utilizes openly accessible datasets to obtain more accurate estimates and increased statistical potency as a result of the extensive sample sizes in GWAS. The findings were not influenced by horizontal pleiotropy or other variables. In summary, this study had sufficient statistical power to identify a significant correlation between gut microbiota/immune cells and MT. However, there were certain limitations that need to be acknowledged. Firstly, in order to minimize the impact of population stratification bias, the majority of participants included in our analysis were of European descent, which could potentially introduce some bias into our findings. Secondly, due to the unavailability of demographic information such as gender and ethnicity in the original dataset, we were unable to conduct subgroup analyses. Thirdly, since the number of SNPs meeting the genome-wide significance threshold (*p* < 5 × 10^−8^) was insufficient for further investigation, we only focused on SNPs reaching locus-wide significance level (*p* < 5 × 10^−5^). These constraints may limit the generalizability of our results and could have potentially affected the accuracy of this study.

## 5 Conclusion

In summary, we have extensively verified the link between gut microbiota/immune cells and MT. Specifically, four bacterial characteristics and seven immune cells exhibited a positive causal relationship with MT, while two other bacterial features and nine immune cells displayed a negative causal association with MT. These microbial strains could serve as innovative biomarkers and offer valuable insights for preventing and treating MT.

## Data availability statement

The datasets presented in this study can be found in online repositories. The names of the repository/repositories and accession number(s) can be found in the article/Supplementary material.

## Author contributions

SH: Conceptualization, Data curation, Formal analysis, Writing – original draft, Writing – review & editing. JiG: Writing – original draft. ZW: Writing – original draft. YX: Writing – review & editing. YG: Writing – review & editing. YL: Funding acquisition, Writing – review & editing. JuG: Conceptualization, Funding acquisition, Writing – review & editing.
